# Single-session transanal minimally invasive surgery (TAMIS) and adjuvant radiotherapy in a patient with three synchronous early rectal adenocarcinomas: a video vignette

**DOI:** 10.1007/s10151-026-03335-3

**Published:** 2026-05-08

**Authors:** Ata Maden, Noam Shussman, Marc Wygoda, Ido Mizrahi

**Affiliations:** 1 Department of General Surgery, Hadassah Medical Center, Jerusalem, Israel; 2 Hebrew University of Jerusalem, Jerusalem, Israel; 3 Department of Oncology, Hadassah Medical Center, Jerusalem, Israel

## Background

Synchronous colorectal adenocarcinomas are uncommon, and three or more distinct primary tumors confined to the rectum are exceedingly rare [1]. Such presentations raise concern for increased tumor burden and nodal involvement, traditionally prompting radical resection at the cost of long-term functional morbidity and permanent stoma [2].

For appropriately staged early lesions, organ-preserving strategies have gained traction. Advances in transanal techniques, particularly transanal minimally invasive surgery (TAMIS) and transanal endoscopic microsurgery (TEM), allow full-thickness excision of early rectal cancers with high R0 resection rates and sphincter preservation, with oncologic safety demonstrated in highly selected T1–T2 tumors, particularly in the context of neoadjuvant or adjuvant therapy [3, 4]. According to the National Comprehensive Cancer Network (NCCN) Guidelines, patients with pT2 or high-risk pT1 tumors after transanal local excision may be managed either with transabdominal total mesorectal excision (TME) or with postoperative chemoradiotherapy as an organ-preserving treatment pathway [5].

## Case overview

Three synchronous early rectal adenocarcinomas were managed in a single session using TAMIS followed by adjuvant radiotherapy, achieving complete clinical remission at 2 years.

Preoperative biopsies were negative for carcinoma, and pelvic magnetic resonance imaging (MRI) overstaged one lesion; TAMIS therefore served both diagnostic and therapeutic purposes through full-thickness excision.

This report describes the multidisciplinary management and 2-year outcome. The accompanying video demonstrates multifocal full-thickness TAMIS resection, achievement of clear margins, and safe closure of multiple rectal wall defects.

## Case presentation

We report the case of a 50-year-old, healthy asymptomatic male, without personal or family history suggestive of hereditary colorectal cancer, who underwent his first screening colonoscopy, revealing three distinct rectal polyps between 4 and 7 cm from the anal verge.

The polyps were characterized as:

- Distal: 40 mm, ulcerated, pedunculated, with spontaneous bleeding.
- Middle: 10 mm, flat, sessile.
- Proximal: 35 mm, narrow-based, pedunculated, with spontaneous bleeding.

Only the distal lesion was biopsied, yielding high-grade dysplasia (HGD).

After the initial community-based colonoscopy, the patient was referred to our institution and underwent a repeat colonoscopy with a colorectal surgeon present. The results, including an additional biopsy from the distal polyp, were unchanged. Computed tomography (CT) of chest, abdomen, and pelvis revealed no metastasis, and pelvic MRI showed no suspicious lymphadenopathy, characterizing the polyps as:

- Distal: no invasion into muscularis propria, cTis/cT1.
- Middle: no invasion into muscularis propria, cTis/cT1.
- Proximal: invasion into perirectal fat ~3–4 mm with evidence of lymphovascular invasion, cT3a.

Following multidisciplinary team (MDT) discussion, upfront transanal local excision was recommended on the basis of concordant clinical findings, including soft and mobile lesions on digital rectal examination and repeat colonoscopy demonstrating feasibility of a transanal approach, despite reviewed MRI findings suggesting cT3a disease. The option of TME was discussed; however, given the distal tumor location with anticipated need for a very low colorectal or coloanal anastomosis and high risk of functional impairment, local excision was selected to initiate treatment and obtain definitive histopathologic staging, recognizing the known potential for pelvic MRI overstaging in early rectal lesions.

All three tumors were resected using TAMIS. The patient was discharged on postoperative day 1 without complications.

Histopathologic examination confirmed all polyps to be malignant:

- Distal: well-differentiated adenocarcinoma, arising in a tubulovillous adenoma (TVA) with HGD; submucosal invasion 2.1 mm (pT1 Sm2).
- Middle: TVA with HGD, foci of intramucosal carcinoma (pTis).
- Proximal: well to moderately differentiated adenocarcinoma, arising in a TVA with HGD; submucosal invasion 2 mm (pT1 Sm2).

All specimens included muscularis propria and had negative margins, without lymphovascular or perineural invasion.

Although each lesion individually fulfilled low-risk criteria, the presence of three synchronous rectal adenocarcinomas with two pT1 Sm2 components was considered biologically distinct from a solitary low-risk T1 lesion. In the absence of high-risk histopathologic features, adjuvant radiotherapy without chemotherapy was selected as a risk-adapted organ-preserving strategy after subsequent MDT discussion.

Long-course pelvic radiation (46 Gy, 23 fractions) was initiated 3 months postoperatively and was completed without complications.

The patient continued follow-up under MDT supervision. At 6 months, carcinoembryonic antigen (CEA) and laboratory studies were normal, and digital rectal examination was unremarkable. At 1 year, CEA, laboratory studies, rectal examination, colonoscopy, pelvic MRI, and positron emission tomography (PET)–CT showed no abnormalities. At 2 years, clinical examination, CEA, laboratory tests, along with colonoscopy, CT of the chest, abdomen, and pelvis remained unremarkable.

Follow-up colonoscopies demonstrated a patent rectal lumen without endoscopic stenosis and were completed using a standard-caliber colonoscope without technical issues.

The patient reported no symptoms of functional impairment during follow-up. Vaizey incontinence score was obtained throughout follow-up for further functional assessment and remained at zero.

This report is limited by its single-patient design, the absence of pathologic nodal staging, and a follow-up duration of 2 years, which may be insufficient to capture late local or distant recurrence. The selected clinical context and favorable tumor biology limit generalizability, and longer follow-up and larger series are required to confirm durable oncologic safety.

The operative technique and case summary are shown in Video Supplementation (figshare link: https://figshare.com/articles/media/Single-session_transanal_minimally_invasive_surgery_TAMIS_and_adjuvant_radiotherapy_in_a_patient_with_three_synchronous_early_rectal_adenocarcinomas_a_video_vignette/31889002).

## Supplementary Information

Below is the link to the electronic supplementary material.

Supplementary file1 (MP4 318116 KB)

## Data Availability

No datasets were generated or analyzed during the current study.

## Abbreviations

TAMIS: Transanal minimally invasive surgery
TEM: Transanal endoscopic microsurgery
TME: Total mesorectal excision
MRI: Magnetic resonance imaging
HGD: High-grade dysplasia
CT: Computed tomography
MDT: Multidisciplinary team
TVA: Tubulovillous adenoma
CEA: Carcinoembryonic antigen
